# Hybrid Funnel Technique Reduces Marginal Bone Loss Versus Conventional Drilling: A 3‐Year, Prospective, Non‐randomized, Controlled Clinical Trial

**DOI:** 10.1111/cid.70175

**Published:** 2026-07-28

**Authors:** Luigi Canullo, Yaniv Mayer, Federica Mattalia, Iole D'Urso, Filiberto Mastrangelo, Vito Carlo Alberto Caponio

**Affiliations:** ^1^ Department of Prosthodontic University of Saint Camillus Rome Italy; ^2^ Department of Periodontology University of Bern Bern Switzerland; ^3^ Ruth and Bruce Rappaport Faculty of Medicine at the Technion Haifa Israel; ^4^ Department of Periodontology School of Graduate Dentistry, Rambam Health Care Campus Haifa Israel; ^5^ Department of Surgical Sciences and Integrated Diagnostics University of Genova Genoa Italy; ^6^ Department of Clinical and Experimental Medicine University of Foggia Foggia Italy; ^7^ Department of Life Sciences, Health and Health Professions Link Campus University Rome Italy

**Keywords:** bioactive implant surface, dental implants, hybrid funnel technique, implant site preparation, marginal bone loss, osseointegration, peri‐implant bone

## Abstract

**Purpose:**

This study aimed to compare marginal bone loss (MBL) associated with Hybrid Funnel Technique (HFT) and conventional drill osteotomy for implant site preparation when bioactive implants are used, testing the null hypothesis that no significant differences occur at 1‐ and 3 years.

**Materials and Methods:**

This prospective non‐randomized controlled clinical trial included patients undergoing implant rehabilitation with bioactive‐surface implants. Implants were placed using either conventional subtractive drilling (control group) or HFT (test group), which combines selective cortical preparation with medullary osteocompaction. The primary outcome was radiographic MBL at 3‐year follow‐up. Secondary outcomes included bleeding on probing (BoP) and plaque index (PI). Statistical analyses were performed at implant level, and linear mixed‐effects models were used to account for clustering within patients and adjust for potential confounders.

**Results:**

A total of 87 implants in 43 patients were analyzed (42 control, 45 HFT). At 1‐year follow‐up, the test group reported significantly lower MBL (0.33 ± 0.43 mm vs. mean = 0.78 ± 0.82 mm; Mann–Whitney *p*‐*value* = 0.006). At 3 years, MBL was significantly higher in the conventional site preparation group compared with the HFT group (1.34 ± 0.79 mm vs. 0.46 ± 0.69 mm; *p* < 0.001). In multivariate mixed‐effects analysis, the implant site preparation technique was the only variable independently associated with MBL at 3 years (*β* = −1.10 mm, 95% CI = −1.675 to −0.527, *p* < 0.001), while insertion torque and other baseline variables showed no significant association. In addition, the HFT group demonstrated significantly lower BoP and PI scores, indicating more favorable peri‐implant soft tissue conditions.

**Conclusions:**

Despite the use of identical bioactive‐surface implants, implant site preparation technique significantly influenced peri‐implant bone remodeling. HFT demonstrated superior preservation of marginal bone over 3 years, suggesting that reduction of cortical compression during osteotomy provides a biological advantage. Implant site preparation remains a key determinant of long‐term peri‐implant bone stability.

## 1 Introduction

Long‐term preservation of peri‐implant marginal bone is considered one of the principal determinants of implant success, contributing to the maintenance of osseointegration, peri‐implant soft tissue stability, and favorable prosthetic outcomes [1]. Among the numerous factors affecting peri‐implant bone remodeling [2], the surgical preparation of the implant site has received increasing attention because it directly influences the mechanical and biological events occurring during the early phases of healing [3].

Various osteotomy protocols have been developed to optimize implant stability according to the characteristics of the recipient bone [4]. Conventional drilling techniques primarily prepare the implant bed through bone removal, whereas alternative approaches, including undersized preparation [5] and osseodensification, aim to preserve or compact bone to enhance primary stability, particularly in sites with reduced bone density [6, 7]. Although these techniques have demonstrated favorable mechanical performance under specific clinical conditions, concerns remain regarding excessive compression of peri‐implant bone and its potential influence on subsequent bone remodeling [3, 8].

Experimental and clinical evidence suggests that cortical and cancellous bone respond differently to surgical manipulation because of their distinct structural and biological characteristics. While controlled condensation of cancellous bone may improve implant stability, excessive compression of cortical bone has been associated with increased remodeling activity and greater crestal bone changes [9, 10]. Consequently, implant site preparation protocols that selectively address the biomechanical behavior of the two bone compartments may represent a biologically sound strategy to optimize both primary stability and peri‐implant bone preservation.

Beyond surgical technique, implant surface topography, at both macro‐ and nanoscopic levels, plays a crucial role in modulating implant stability. Surface characteristics influence primary stability by increasing bone‐to‐implant contact and by affecting biological stability by regulating protein adsorption, cell adhesion, and cell‐to‐cell interactions [11, 12]. It has been shown that surface roughness, super‐ and hydrophilicity, and wettability significantly enhance osseointegration by promoting the adhesion, proliferation, and differentiation of osteogenic cells [13, 14]. Based on this biological rationale, it could be hypothesized that the biological advantages of bioactive implants surfaces may reduce the need for a specific implant site preparation protocol. The enhanced cellular recruitment and biological responsiveness of bioactive surfaces may offset the adverse effects of a greater mismatching.

The Hybrid Funnel Technique (HFT) was developed based on combining conventional drilling for cortical bone preparation with controlled condensation of the cancellous compartment. A preliminary clinical investigation reported encouraging short‐term outcomes, including favorable marginal bone preservation and peri‐implant tissue stability. However, evidence regarding the medium‐term clinical performance of this approach remains limited, particularly when bioactive implants are considered [15].

Therefore, the aim of the present prospective non‐randomized controlled clinical study was to compare peri‐implant marginal bone loss (MBL) after 3 years between implants placed using the HFT and those placed following a conventional drilling protocol. The null hypothesis was that no significant differences would be observed between the two implant site preparation techniques with respect to MBL when bioactive implants are employed.

## 2 Materials and Methods

### 2.1 Study Design and Patient Selection

The present study was designed as a prospective non‐randomized controlled clinical trial with the aim of comparing MBL associated with two different implant site preparation techniques, namely the HFT and conventional drill osteotomy implant site preparation, for rehabilitation with bioactive‐surface implants. The study included a clinical and radiographic follow‐up period of 3 years. The study was reported according to STROBE guidelines [16].

Patients undergoing implant‐prosthetic rehabilitation in the maxillary and/or mandibular regions at a private dental clinic in Rome, Italy, were included. Patients were consecutively recruited between July 2022 and November 2023. Clinical and radiographic data were prospectively collected as part of routine clinical practice, according to a predefined follow‐up schedule. Non‐randomized allocation was adopted.

All clinical procedures were performed in accordance with the principles of the Declaration of Helsinki and were approved by the local Ethics Committee (Comitato Etico del Lazio I, submission protocol #745/CE Lazio 1—approved protocol #904/CE Lazio 1–7 July 2022). The study protocol was approved by the Institutional Review Board before patient recruitment commenced. All patients were adequately informed about the objectives and procedures of the study and provided written informed consent prior to inclusion and before the execution of any clinical procedure. The study was registered in the ISRCTN registry (ISRCTN81043520; DOI: 10.1186/ISRCTN81043520—Date of first enrolment 29 July 2022), recognized by the World Health Organization and the International Committee of Medical Journal Editors that accepts all clinical research studies to promote transparency. The study was registered retrospectively due to administrative oversight.

Inclusion and exclusion criteria are listed in Table 1. Only patients presenting satisfactory periodontal conditions (full mouth PI and BoP < 25%) were included in the study, and all participants were enrolled in a regular supportive periodontal maintenance program throughout the follow‐up period to maintain comparable oral hygiene conditions. Plaque accumulation and soft tissue health were assessed at six sites per tooth/implant using the PI described by Silness and Löe and the Modified Sulcus Bleeding Index [17].

**TABLE 1:** Inclusion/exclusion criteria.

Inclusion criteria
Patients requiring implant rehabilitation in the maxillary and/or mandibular bone
ASA physical status I–II
Age between 30 and 85 years
Healthy or treated periodontal conditions (treated periodontitis, PI < 25%, BoP < 25%)
Willingness to sign the informed consent and to participate in the clinical study

Exclusion criteria
Absence of type 1–2 post‐extraction sites
ASA physical status III–IV
Untreated periodontitis
Sites with a history of previous implant failure
Known allergies to one or more medications used during treatment
Pregnancy (confirmed by verbal inquiry)

### 2.2 Implant Characteristics, Group Allocation, and Surgical Procedure

All implants used in the present study were NINA MultiNeO NH implants (Alpha‐Bio Tec), characterized by a bioactive surface obtained through a chemical–physical treatment involving sandblasting with aluminum oxide, double acid etching, and subsequent surface modification resulting in the formation of a titanium oxide nano‐topography.

The hydrophilic component of the surface is maintained by the presence of a resorbable salt layer. The coronal implant design is characterized by the presence of exclusive “coronal cutting flutes,” which are intended to significantly reduce crestal stress, thereby promoting preservation of bone volume and optimal aesthetic outcomes.

Eligible patients were prospectively assigned before surgery to one of the two treatment groups according to a predefined non‐randomized allocation protocol. Allocation was performed with the objective of obtaining clinically comparable groups regarding age, sex, implant site, bone density, and cortical bone characteristics, while maintaining the feasibility of the clinical protocol. All treatments were performed by a single experienced operator (LC).

During the preoperative phase, patients underwent clinical and radiographic evaluation, including intraoral photographs, intraoral scanning, and periapical radiographs. A cone‐beam computed tomography (CBCT) scan was also performed for anatomical assessment and treatment planning and was subsequently used as a reference for radiographic evaluations. All patients received a full‐mouth professional oral hygiene session and oral hygiene instructions at least 2 weeks prior to surgery. Antibiotic prophylaxis with amoxicillin/clavulanic acid was prescribed.

Surgical planning was carried out using dedicated computer‐guided surgery software (3DM, Milan, Italy) based on CBCT images; once planning was completed, an individualized surgical guide was fabricated by a dental laboratory.

All surgical procedures were performed under local anesthesia using lidocaine with epinephrine 1:100000. A small mucoperiosteal flap was elevated and, using the surgical guide, implants were placed according to preoperative planning. During surgery, cortical bone thickness was measured and recorded using a calibrated periodontal probe (PCP‐15 UNC). All implants were placed approximately 0.5 mm below the crestal bone level according to the study protocol to standardize implant positioning between groups.

Implant site preparation was performed according to two different protocols. In the control group (conventional technique), the osteotomy was prepared using a standard sequence of subtractive drills (Coated Straight Drills 4550‐4555, Alpha‐Bio Tec) in accordance with the manufacturer's instructions, including a 2.0‐mm pilot drill to the planned implant length, a 3.0–2.0 mm step drill, and a final 3.2‐mm drill to the full implant length, followed by implant insertion.

In the test group, implant site preparation was performed using the HFT, which combines subtractive and non‐subtractive principles. A 2.0‐mm pilot drill and a 3.0–2.0 mm step drill were first used to the planned implant length; subsequently, selective preparation of the crestal cortical bone was performed using a 3.65‐mm drill, while the medullary portion was condensed using a dedicated osteotome (Umberto Merighi, Rimini, Italy) corresponding to the implant body diameter prior to implant insertion. This approach results in a funnel‐shaped osteotomy configuration, characterized by reduced compression of the coronal cortical bone and selective engagement of the medullary compartment to achieve primary stability.

In both groups, insertion torque values were recorded at the time of implant placement. A baseline periapical radiograph was obtained at the end of the surgical procedure. Primary wound closure was achieved using 6‐0 sutures (Monofast, Medipac, Thessalonica, Greece). Postoperative management included systemic antibiotic therapy, anti‐inflammatory medication, and saline mouth rinses for 10 days; patients were also instructed to follow a soft diet and apply cold packs. Sutures were removed after 14 days.

Patients were enrolled in a clinical and radiographic follow‐up program and supportive periodontal therapy for the entire duration of the study. Follow‐up visits were scheduled at regular intervals of 3–6 months according to routine clinical practice, with particular attention to the monitoring of peri‐implant and adjacent dental tissues, assessment of oral hygiene, and reinforcement of motivation when necessary. Clinical and instrumental evaluations were performed during the early postoperative period and subsequently at 6 and 12 months, including periapical radiographs and, when indicated, three‐dimensional imaging (CBCT). After the first year, patients continued periodic follow‐up visits until the final 3‐year follow‐up, which was used for the assessment of the study outcomes. To minimize prosthetic‐related variability, all implants were restored according to a standardized loading protocol consisting of cement‐retained single crowns delivered approximately 3 months after implant placement.

### 2.3 Outcomes and Radiographic Evaluation

The primary endpoint of the present study was MBL assessed at the 3‐year follow‐up, which was considered a clinically relevant indicator of the biological response of peri‐implant bone to the different implant site preparation techniques. Secondary endpoints included bleeding on probing (BoP) and plaque index (PI), which were used to monitor peri‐implant soft tissue health and oral hygiene conditions throughout the follow‐up period.

Radiographic evaluation was performed using standardized periapical radiographs with parallel technique with standard holders acquired at the time of implant placement (baseline), at 1‐ and at the final 3‐year follow‐up visit. Radiographic images were used for the assessment of MBL, calculated as the change in marginal bone level measured at the mesial and distal aspects of each implant between the different time points. Measurements were performed using reproducible implant‐related anatomical reference points to ensure comparability over time and to minimize potential bias related to radiographic variability. The mean value of the mesial and distal measurements was subsequently used for statistical analysis. All radiographic measurements were performed by a calibrated examiner on image processing software ImageJ 1.51 (https://imagej.net/ij/docs/index.html), who was blinded to treatment allocation. Examiner calibration was conducted by repeating measurements on a representative sample of 20 radiographs before the final analysis. The examiner repeated the measurements after a 2‐week interval. Intra‐examiner reliability was excellent, with an intraclass correlation coefficient (ICC, two‐way mixed‐effects model, absolute agreement) of 0.99%.

### 2.4 Sample Size Calculation

To our knowledge, this is the first study comparing HFT and conventional implant site preparation techniques, employing only bioactive implants. Therefore, sample size was estimated a priori based on the results from Trombelli et al. [18]. This study found a mean annual rate of change in averaged marginal bone level (mesial and distal sites) of approximately 0.15 ± 0.8 mm. Since in this study, a 3 year follow‐up endpoint was considered, a between‐group difference of 0.5 mm in mean MBL at 3 years was considered clinically relevant, as it exceeds radiographic measurement error and physiological remodeling and reflects bone changes of a magnitude observable at the individual implant level. Sample size was estimated in G*Power 3.1.9.7, assuming an effect size of 0.625, a two‐sided significance level of 0.05, and a statistical power of approximately 80%. Effect size was estimated according to Cohen's *d* formula Δ/common standard deviation (0.50/0.8). Estimation resulted in 42 implants per group.

### 2.5 Statistical Analysis

All the analyses were performed at implant level. Statistical analysis was performed blindly to study design and settings. To explore baseline differences between the two treatment groups, *χ* ^2^ test was used for dichotomous clinical and implant characteristics. Normality of continous variables was assessed by Shapiro–Wilk test, therefore, parametric or non‐parametric analysis test was used to inspect differences in means at baseline (independent‐samples *t*‐test or Mann–Whitney or Kruskal‐Wallis test). To investigate which baseline variables were associated with MBL at 3 years (dependent variable), a linear mixed‐effects model was used. Because multiple implants were placed in the same patient, patient ID was included as a random intercept to account for within‐patient correlation. Fixed effects included treatment group and clinically relevant baseline patient, implant, and bone‐related variables. Statistical analyses were performed using SPSS (v25) and R (v4.4.1).

## 3 Results

### 3.1 Study Population and Baseline Characteristics

**FIGURE 1:**
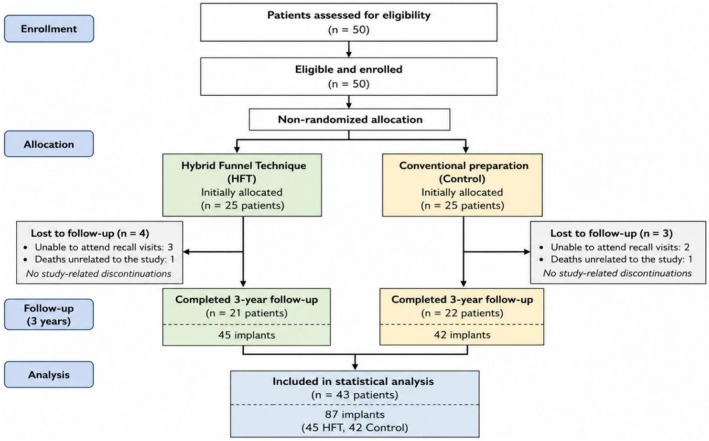
Flow diagram of the recruitment and analysis pipeline.

A total of 50 patients were initially allocated to the test group (HFT) and the control group (conventional implant site preparation). Of these, 43 patients completed the follow‐up at 3 years. Overall, a total of 87 implants were included in the analysis (42 in the control and 45 in the test group). Patients lost to follow‐up were due to inability to attend recall visits (*n* = 5) and death unrelated to the study (*n* = 2); no study‐related complications led to study discontinuation. Each patient received a mean number of 2 implants. Once the estimated sample size was reached, data were analyzed without those patients who did not yet complete the 3‐year follow‐up period (Figure 1).

Continuous variables showed a non‐normal distribution (Shapiro–Wilk *p*‐*value* < 0.001). Most baseline characteristics were comparable between the treatment groups. A significantly higher proportion of implants were placed in the mandible in the conventional group (*χ* ^2^ test *p*‐*value* = 0.007), which may explain why implant insertion torque was higher in this group (Mann–Whitney *p*‐*value* < 0.001), mean = 54.24 (13.85), vs. 30.62 (9.75) (Table 2).

**TABLE 2:** Distribution of clinical variables at baseline between test and control.

Variable list		Treatment group								
		Control				Test				*p*
		Count	Row *N* %	Mean	Standard deviation	Count	Row *N* %	Mean	Standard deviation	
Sex	Male	23	57.50%			17	42.50%			0.112
	Female	19	40.40%			28	59.60%			
Implant location	Maxilla	15	34.10%			29	65.90%			**0.007**
	Mandible	27	62.80%			16	37.20%			
Age				62	10			54	14	0.08
Gingival height (mm)				3.18	0.63			3.16	0.56	0.757
Cortical thickness (mm)				0.4	0.6			0.5	0.6	0.687
Implant diameter (mm)				3.78	0.2			3.78	0.22	0.962
Implant length (mm)				9.5	1			9.7	0.92	0.214
Implant insertion torque				54.24	13.85			30.62	9.75	**< 0.001**
Bone density (HU)				594	261			581	268	0.802

*Note:* Bold = *p* < 0.05.

### 3.2 Clinical Outcomes

At 1‐year follow‐up, the HFT group reported significantly lower MBL (mean_control_ = 0.78 mm, SD: 0.82 mm, vs. mean_HFT_ = 0.33 mm, SD: 0.43 mm, Mann–Whitney *p*‐*value* = 0.006).

**FIGURE 2:**
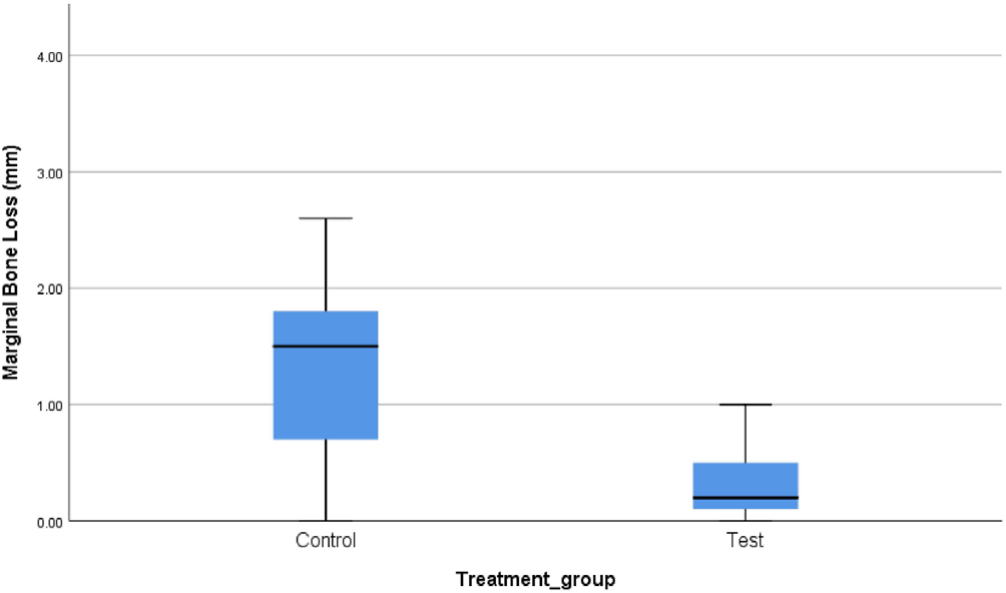
Marginal bone loss at 3‐year follow‐up in the control vs. test group.

At the 3‐year follow‐up, MBL was significantly higher in the control group compared to the HFT group (mean = 1.34 mm (SD: 0.79 mm), vs. 0.46 mm (SD: 0.69 mm), Mann–Whitney *p*‐*value* < 0.001) (Figure 2); as well as the conventional group showed higher mean values in BOP and PI (Mann–Whitney *p*‐*value* < 0.001) (mean_BOP_ = 1.53, SD: 0.78, vs. 0.73, SD: 0.61; mean_PI_ = 1.33, SD: 0.65, vs. 0.47, SD: 0.66).

Variance inflation factors ranged from 1.08 to 2.35, for this reason all the variables were retained in the regression model. In the linear mixed‐effects model accounting for clustering within patients (random intercept for Patient ID), treatment group was the only significant independently associated factor of 3‐year MBL. Implants in the test group exhibited significantly lower bone loss compared with the control group (*β* = −1.10, 95% CI = −1.675 to −0.527, *p*‐*value* < 0.001), after adjusting for age, sex, jaw, bone density, cortical thickness, implant insertion torque, implant diameter and length, and gingival height. None of the other baseline patient‐ or implant‐level variables were significantly associated with MBL. The variance attributable to patient‐level differences was 0.19 mm^2^, corresponding to an adjusted intraclass correlation of 0.44, indicating that 44% of the variability in MBL was due to differences between patients (Table 3).

**TABLE 3:** Linear mixed‐effects model of baseline patient‐ and implant‐level independently associated factors of 3‐year MBL.

Variable	*β* (mm)	SE	*t*	*p*	95% CI (mm)
Treatment group (Test vs. Control)	−1.10	0.29	−3.76	0.0007	−1.675 to −0.527
Age (scaled)	0.089	0.102	0.878	0.39	−0.110 to 0.289
Sex (Male vs. Female)	−0.226	0.209	−1.08	0.290	−0.635 to 0.183
Implant location (Mandible vs. Maxilla)	−0.282	0.245	−1.14	0.262	−0.764 to 0.199
Bone density (HU) (scaled)	−0.076	0.081	−0.93	0.354	−0.236 to 0.084
Cortical thickness (mm)	0.06	0.15	0.389	0.699	−0.239 to 0.358
Implant diameter (mm)	0.02	0.364	0.055	0.956	−0.694 to 0.734
Implant insertion torque (N)	−0.001	0.007	−0.22	0.822	−0.015 to 0.012
Implant length (mm)	0.05	0.085	0.594	0.554	−0.116 to 0.218
Gingival height (mm)	0.025	0.19	0.132	0.895	−0.350 to 0.400

*Note:* Regression coefficients (β), standard errors (SE), *t*‐values, *p*‐values, and 95% confidence intervals (CI) were included. Patient ID was included as a random intercept to account for clustering of multiple implants within the same patient.

Similarly, at 1‐year follow‐up, a linear mixed‐effects model accounting for clustering within patients showed that treatment group was significantly associated with MBL. Implants in the test group exhibited lower MBL compared with the control group (*β* = −0.72 mm; *p‐value* = 0.027), after adjustment for age, sex, jaw, bone density, cortical thickness, implant insertion torque, implant diameter and length, and gingival height. None of the other baseline patient‐ or implant‐level variables were significantly associated with 1‐year MBL. The variance attributable to patient‐level differences was 0.30 mm^2^, indicating substantial between‐patient variability.

## 4 Discussion

In the present study, the HFT was associated with significantly lower MBL at 1‐ and 3‐year follow‐ups compared with conventional implant site preparation, with an approximately 1‐mm reduction in MBL. This difference exceeds the threshold of physiological remodeling and radiographic measurement error, supporting its clinical relevance. The difference remained statistically significant after adjustment for patient‐ and implant‐related variables in a linear mixed‐effects model, indicating that the observed effect was primarily attributable to the implant site preparation technique itself.

The reduced MBL observed in the HFT group may be explained by the different biomechanical environment generated at the crestal cortical level. Unlike conventional or undersized preparation protocols, the HFT is designed to limit lateral compression of the cortical bone while still achieving adequate primary stability through selective engagement of the medullary compartment.

Numerous clinical and experimental studies have demonstrated that cortical compression during implant insertion may result in microfractures, microcirculatory alterations, and compression necrosis, subsequently triggering marginal bone resorption [19, 20]. In this context, early MBL has been described as a non‐infectious bone response to surgical trauma, particularly when excessive mechanical or thermal stresses affect the cortical bone [21]. The higher mean values of MBL, BOP, and PI observed in the conventional preparation group in the present study are consistent with this biological interpretation, suggesting a less favorable peri‐implant tissue response over time.

Consistently, the present statistical analysis showed that the treatment group remained the only significant independently associated factor of MBL at 3 years, while implant insertion torque, although generally higher in the conventional group, was not significantly associated with bone loss. This finding aligns with growing evidence indicating that achieving high primary stability values, often pursued through undersized preparation protocols, does not necessarily translate into improved biological outcomes. Recent studies have reported a tendency toward greater MBL in underprepared sites compared with conventionally prepared sites, particularly in the presence of cortical bone (D1–D3), whereas in D4 bone the mismatch between implant and osteotomy appears to have a clinically negligible impact on MBL [10]. Overall, these results support the need for biologically driven rather than purely mechanical surgical strategies.

Within this framework, HFT was developed as an innovative approach to implant site preparation combining principles of ablative osteotomy and osteocompaction. HFT is designed to adapt the osteotomy protocol to the bone quality of the recipient site through differential management of the cortical and medullary compartments. This approach allows exploitation of the elastic properties of cancellous bone to achieve predictable primary stability, while simultaneously reducing the risk of biological damage to the cortical bone, such as thermal friction necrosis or microfractures. To this end, the cortical component is prepared using dedicated ablative instruments, including drills or piezoelectric tips, to selectively remove bone and avoid excessive compression, whereas the medullary portion is subsequently compacted using manual osteotomes, promoting a biological environment favorable to faster secondary stability mediated by intramembranous ossification. From a geometric perspective, HFT reproduces a funnel‐shaped osteotomy, characterized by a wider diameter in the coronal portion corresponding to cortical thickness and a more conservative, compacting preparation in the apical medullary portion [15].

This configuration is consistent with experimental evidence indicating that osteotomy design influences peri‐implant bone remodeling. Excessive cortical compression may delay osseointegration due to the need to resorb necrotic tissue prior to new bone deposition, whereas an osteotomy diameter comparable to that of the implant promotes the formation of healing chambers, allowing faster new bone formation and earlier secondary stability [22, 23].

The clinical outcomes observed in the present study are in line with previous investigations introducing the HFT technique as an alternative preparation protocol. A retrospective clinical study evaluating HFT reported complete implant osseointegration at 12 months, limited MBL (0.17 ± 0.21 mm), and favorable aesthetic outcomes, indicating stable peri‐implant tissues [15]. Although the absolute MBL values were slightly higher in the current study, this difference is likely attributable to differences in study design, patient population, sample size, and the inclusion of a comparative control group. Importantly, despite these methodological differences, both studies consistently demonstrated limited peri‐implant bone remodeling associated with the HFT. Furthermore, the present study extends the evidence by demonstrating that this favorable effect was maintained after 3 years, with significantly lower MBL in the HFT group compared with the conventional drilling protocol. Conversely, partially different results have been reported in experimental animal models: in a rabbit study comparing conventional drilling and HFT preparation with osteotomes, no significant differences in MBL or cervical bone‐to‐implant contact were observed, regardless of bone density or healing time [24].

It is also important to consider that all implants used in the present study were characterized by bioactive surfaces. Bioactivation of titanium surfaces has been widely demonstrated to modify their physicochemical properties by increasing wettability and surface energy, promoting early protein adsorption, and enhancing cellular adhesion, proliferation, and organization [25, 26]. These modifications translate into a more efficient biological response of peri‐implant tissues, characterized by faster cellular recruitment and more organized early healing [27, 28]. In this context, it was hypothesized that the use of bioactive implant surfaces could at least partially attenuate local cellular damage induced by surgical trauma. Nevertheless, despite the use of identical bioactive surfaces in both groups, the implant site preparation technique remained the sole significant independently associated factor of MBL, indicating that surface bioactivation alone is not sufficient to offset the negative biological effects associated with excessive cortical compression.

From a clinical perspective, the results of the present study suggest that implant site preparation should be customized according to bone density and the histological compartment involved, rather than relying exclusively on undersizing strategies or the achievement of high insertion torque values. In sites characterized by thick and dense cortical bone, techniques aimed at reducing cortical stress may be particularly advantageous for the preservation of marginal bone.

The limitations of the present study include the relatively limited sample size, the non‐randomized study design, and the absence of histological or biomechanical evaluations. Although treatment allocation was planned prospectively and multivariable mixed‐effects models were used to adjust for measured baseline characteristics, residual selection bias and unmeasured confounding cannot be completely excluded. In addition, peri‐implant marginal bone remodeling is recognized as a multifactorial process influenced by biological [29], surgical, prosthetic, anatomical [2], and clinician‐ [30] and patient‐related factors [31, 32]. To minimize potential confounding, implant placement depth, prosthetic abutments, loading protocol, and oral hygiene maintenance were standardized across participants, while baseline bone characteristics and implant location were considered during treatment allocation and statistical analysis. The baseline imbalance in implant location between the study groups should also be considered when interpreting the findings. Implant location is known to influence peri‐implant bone remodeling through differences in anatomical characteristics and bone quality. To reduce this potential source of confounding, implant location, bone density, and cortical thickness were included as covariates in the multivariable mixed‐effects analysis. Nevertheless, residual confounding related to anatomical site or from unmeasured variables, including individual host response, occlusal loading patterns, and other patient‐related factors, cannot be completely excluded. Consequently, the present findings should be interpreted with appropriate caution and confirmed by larger randomized controlled trials with longer follow‐up periods. Another limitation of the present study is the retrospective registration of the clinical study. Although the study protocol, eligibility criteria, primary outcome, and follow‐up schedule were established before patient enrollment and remained unchanged during the study, retrospective registration is less transparent than prospective registration and may raise concerns regarding outcome reporting bias.

However, another aspect to consider is the limited number of clinical studies currently available in the literature on the HFT, which makes extensive and systematic comparisons with consolidated data difficult. The 3‐year follow‐up period and the use of multivariate statistical models strengthen the reliability of the observed associations. Future studies with larger cohorts, longer observation periods, and multidisciplinary assessment protocols are warranted to further validate these findings. Furthermore, although BoP and PI were evaluated as secondary outcomes, other clinically relevant parameters, such as mucosal recession, patient‐reported outcome measures, and implant stability assessments, were not investigated. Future prospective randomized studies incorporating these clinical outcomes would provide a more comprehensive evaluation of the biological and functional performance of the different implant site preparation techniques.

## 5 Conclusions

Within the limits of the present controlled clinical trial, HFT was associated with significantly lower MBL at 3 years compared with conventional implant site preparation, despite the use of identical bioactive surfaces in both groups. These results support the concept that reducing cortical compression during osteotomy positively influences peri‐implant bone behavior, and that implant site preparation remains a critical determinant of long‐term biological outcomes.

In light of these considerations, the findings of the present study have relevant clinical implications, supporting the adoption of biologically oriented and compartment‐specific implant site preparation protocols as part of a personalized surgical approach in implant dentistry.

## Author Contributions

**Luigi Canullo:** conception, design, clinical work, data collection, drafting, and critical revision of the article. **Yaniv Mayer:** critical revision of the article. **Iole D'Urso:** data collection and drafting of the article. **Federica Mattalia:** data interpretation and drafting of the article. **Filiberto Mastrangelo:** critical revision of the article. **Vito Carlo Alberto Caponio:** statistics, drafting, and critical revision of the article. All authors have read and agreed to the published version of the manuscript.

## Funding

This research was conducted with the support of Alpha‐Bio Tec Ltd. The sponsor did not have any involvement in study design, data analysis, nor manuscript preparation.

## Conflicts of Interest

The authors declare no conflicts of interest.

## Data Availability

The data that support the findings of this study are available from the corresponding author upon reasonable request.
